# Chondroitin Sulfate Supplements for Osteoarthritis: A Critical Review

**DOI:** 10.7759/cureus.40192

**Published:** 2023-06-09

**Authors:** Rui Brito, Diogo Costa, Carina Dias, Patrícia Cruz, Paula Barros

**Affiliations:** 1 Physical Medicine and Rehabilitation, Centro Hospitalar e Universitário de Santo António, Porto, PRT

**Keywords:** joint health, chondroprotection, supplementation, osteoarthritis, chondroitin sulfate

## Abstract

Over the years, chondroitin sulfate (CS) has been used as a slow-acting drug for the treatment of osteoarthritis, for the reduction of pain and improvement of function, and for its disease-modifying properties by limiting cartilage volume loss and joint space narrowing progression. However, there have been inconsistencies in published trials regarding clinical efficacy, with reports of a lack of significant effects compared to placebo. The therapeutic effects of chondroitin sulfate may depend on many variables, such as the source of origin, purity, and contamination with by-products. Another source of confusion may be related to the fact that CS is commonly combined with glucosamine, which makes it challenging to isolate the specific contribution of chondroitin to the therapeutic outcome. This is aggravated by the fact that CS supplements, used in many countries, are not regulated, and labels wrongly claim high levels of purity. Many of these inferior CS products may have been used in clinical trials, which may have had limited but significant results. This has led to recent recommendations to opt for higher-purity pharmacologic-grade CS for the treatment of OA. This article aims to provide an up-to-date view of the current literature regarding the biological effects and efficacy of CS and discusses the quality of available chondroitin sulfate supplements and the current direction in CS investigation. This review concludes that pharmacologic-grade CS supplements may have clinically significant benefits when properly standardized; however, high-quality evidence from properly designed clinical trials is still needed to draw definitive conclusions about clinical efficacy in osteoarthritis.

## Introduction and background

Osteoarthritis (OA) is a common condition that causes serious disability. OA affects approximately 3.5% of the global population. Risk factors for OA include age, female gender, obesity, mechanical factors, injuries, and muscle weakness. It is a condition characterized by progressive loss of joint cartilage and subchondral bone lesions [1]. These changes lead to pain, stiffness, and loss of function [2,3]. Muscle weakness and balance changes may also occur [4,5].

OA affects synovial joints, which are made of a capsule lined by the synovium and cartilage that covers bone ends. Cartilage is composed of water, chondrocytes, collagen, and proteoglycans. Damage to the articular cartilage is a characteristic feature of OA. Slow depletion of collagen and proteoglycans in the cartilage leads to catabolism of the collagen network [6]. This causes chondrocytes to proliferate and form clusters. Chondrocyte hypertrophy occurs, leading to the formation of osteophytes. In this process, many inflammatory mediators might be involved. [7-9]

Chondroitin sulfate (CS) is a natural glycosaminoglycan and is found in all connective tissues, especially in the extracellular matrix (ECM) of articular cartilage. This sulfate is covalently attached to a sugar composed of glucuronic acid (GlcA) and N-acetylgalactosamine (GalNAc). CS possesses a negative charge, interacts with proteins in the ECM, and helps regulate many cellular processes [10].

CS belongs to the oral SYSADOA (symptomatic slow-acting drugs for osteoarthritis) with the ability to slow down the progression of osteoarthritis, as has been demonstrated in various trials [11,12]. The substance has a delayed onset of action, and its therapeutic effect can only be accessed after a few weeks of treatment. CS has also been studied as a DMOAD (disease-modifying OA drug) with the ability to modify the course of the OA disease and its progression by decreasing joint space narrowing progression [11,13]. 

Over the years, many studies have demonstrated the beneficial effects of CS on OA [14-20]; however, there has also been conflicting evidence in some published trials [21-24], which leads to a constant debate regarding the role of CS in the treatment of osteoarthritis.

Due to these inconsistencies in older clinical trials and concerns over supplement quality, an up-to-date review of CS for OA is needed to evaluate its therapeutic potential and provide guidance for future research. This article aims to review the biological effects of CS that may be important for the promotion of joint health, discuss the different formulations of CS and how they may impact clinical trials, and provide insight into the current investigation being performed for its role in the treatment of OA.

## Review

Clinical evidence for CS effects

Chondrocytes are essential for the preservation of the normal characteristics of the cartilage. In OA cartilage, apoptotic chondrocytes were found in higher numbers than in normal cartilage [25-27]. This suggests that apoptosis is a potential mechanism for the loss of chondrocytes in OA and progressive cartilage degeneration. CS has been shown to reduce the number of apoptotic chondrocytes in studies in vitro and in vivo [28-30].

Caraglia et al. [28] demonstrated that the addition of mud-sulfur thermal therapy to CS supplementation had improved chondroprotective effects, possibly by potentiating its antioxidant effect.

CS has also demonstrated the ability to increase the production of proteoglycans and type II collagen (anabolic effect) [31-33]. Bassleer et al. [32] demonstrated that CS counteracted the negative effect of IL-1β by increasing ECM components such as proteoglycan and collagen type II and decreasing PGE2 synthesis.

Uebelhart et al. [33] injected chymopapain into rabbit knees, which caused the loss of articular cartilage proteoglycans. Posteriorly, CS was given by oral and intramuscular routes, which resulted in a significant increase in the synthesis of proteoglycan components and demonstrated a protective effect of CS on damaged cartilage.

CS might also have an anti-catabolic effect by limiting the synthesis/activity of metalloproteases, which are responsible for the degradation of ECM components [34-37].

Wang et al. [34] cultivated chondrocytes in media supplemented with IL-1β, which decreased the accumulation of aggrecan, hyaluronan, and type II collagen in the cell-associated matrix (CAM) and increased intracellular MMP-1, -3, and -13. Chrondroitin polysulfate restored the expression of these CAM molecules in these IL-1 beta-treated cultures. This effect probably resulted, in part, from the downregulation of MMPs.

Chou et al. [35] showed that the administration of dietary bars supplemented with CS to rats prevented the raised levels of joint metalloproteases associated with arthritis and prevented cartilage damage.

Chan et al. [36] also demonstrated that cartilage explants exposed to media enriched with IL-1β and CS had decreased expression of IL-1β-induced metalloproteases.

Holzmann et al. [37] provided evidence that CS modulates signaling events in chondrocytes concurrently with MMP-13 downregulation.

CS also plays a role in the modulation of inflammation. Studies have demonstrated that IL-1β is the cytokine responsible for the degradation of ECM components in the tissues of patients with OA [38]. IL-1β activates erk1/2, p38MAPK, and JNK and induces nuclear translocations of NF-κB and AP-1 (activator protein-1), which enhance the expression of pro- and anti-inflammatory cytokines and various enzymes that will perpetuate the inflammatory reaction of OA [39]. CS of bovine origin has been shown to diminish NF-κB nuclear translocation, p38MAPK, and erk1/2 phosphorylation induced by IL-1β [30].

Thus, it appears that nuclear translocation of NF-kB is important in the appearance and perpetuation of synovitis. However, CS has been shown to reduce synovitis in both animals and humans [21,40].

At last, studies have demonstrated that CS may have antioxidant and anti-angiogenic properties [41,42]. In particular, Lambert et al. [42] demonstrated that inflammatory areas of osteoarthritic synovial membranes produced more IL-6, IL-8, and VEGF but less TSP-1 (thrombospondin-1) than cells isolated from normal synovial biopsies. The expression of pro-angiogenic factors by synovial fibroblast cells was stimulated by IL-1β, and anti-angiogenic factors were inhibited. CS reversed the inhibitory effect of IL-1β on anti-angiogenic factors (VEGI [vascular endothelial growth inhibitor] and TSP-1).

Table 1 summarizes the biological effects of CS in OA.

**Table 1:** Chondroitin sulfate effects on osteoarticular tissues GAG: glycosaminoaglycans; HA: hyalouronic acid; TIMP: tissue inhibitor of metalloproteinase; MMP: metalloproteinase; ROS: reactive oxygen species; RANKL: receptor activator of nuclear factor kappa-Β ligand; JNK: c-Jun N-terminal kinase; PGE2: prostaglandin E2; Erk: extracellular signal-regulated kinases

Stimulates	Inhibits
↑ Proteoglycans and GAG	↓ IL-1β
↑ Collagen type 2	↓ PGE2
↑ HA	↓ p38 MAPK, JNK, Erk1/2
↑ TIMP	↓ MMP
↑ Anti-angiogenic factors	↓ NF-κB translocation
↑ Osteoprotegerin	↓ RANKL
	↓ ROS

Issues with supplement quality

In Europe, highly purified pharmaceutical-grade CS products are available as prescription drugs; however, in many countries, such as the United States, CS is only available as a food supplement.

CS supplements vary in terms of origin, quantity, purity, contaminants, and manufacturing practices [43]. There is also a tendency to combine CS supplements with other ingredients, whereas most clinical studies have focused on single-ingredient CS products. CS supplements are not subjected to strict regulatory practices that ensure the quality and purity of prescription products, and some studies have even demonstrated that many do not contain the amount advertised on the label [44].

da Cunha et al. [45] demonstrated that the content of CS in food supplements conformed to the label specifications in less than half of the samples, and the percentages contained in the other samples were very inferior to the percentage declared on the label. The origin of CS contained in food supplements was also variable, with most being of porcine and bovine origin, but cartilaginous fish were also found, and in one sample, the origin could not be determined due to the very low CS content.

Even among pharmaceutical-grade CS, the composition of CS preparations does not always correspond to the manufacturers' description. In fact, one study demonstrated that out of 16 samples, only 5 contained more than 90% CS, while 11 contained less than 15% CS, with maltodextrin being the main contaminant [46].

As such, especially due to quality concerns regarding CS product manufacturing, current recommendations suggest that only pharmaceutical-grade CS formulations should be used in clinical practice [47].

This statement is supported by the fact that a recent meta-analysis demonstrated that when only pharmaceutical-grade CS studies were used, therapeutic effects appeared to be more significant [17]. The smaller standardized mean difference results for CS food supplements might have been affected by the use of the lesser-quality CS preparations mentioned above.

In a recent study [47] comparing pharmaceutical-grade CS versus food supplements (FS), it was demonstrated that only one FS sample (out of 5) had biological effects similar to the pharmaceutical-grade products. This shows that FS efficacy might be limited by the low quality of the chosen FS samples. However, it also demonstrates that high-quality FS may have equivalent therapeutic effects to their pharmaceutical-grade counterparts.

Despite this, because many of these supplements have lower levels of purity, it is recommended that when a CS supplement is considered a therapeutic option, only pharmaceutical-grade CS formulations should be used.

CS as an SYSADOA and DMOAD 

CS is commonly referred to as a SYSADOA and is widely used in the management of OA patients to improve pain and function. Regarding the evidence of clinical efficacy, a recent meta-analysis [18] demonstrated a superior effect of CS against placebo in the reduction of pain and a large effect on the improvement of knee function, as measured by the Lequesne Index. However, there was a high level of inconsistency in clinical trial results due to the risk of bias, study size, and different CS brands. This was already demonstrated in the 2015 Cochrane review [48], which revealed that CS was better than placebo at improving pain and quality of life in patients with OA. Another meta-analysis published in 2019 [49] concluded that CS had small to moderate effectiveness in reducing OA-related pain, with larger dosages (1200 mg/d) having greater benefits than smaller dosages. However, this meta-analysis concluded that CS had only a minimal effect on joint space narrowing and no effect on cartilage volume.

Contrarily, some trials have shown the positive effects of CS as a DMOAD. An RCT [50] demonstrated a reduction in cartilage volume loss at six months and subchondral bone marrow lesions at 12 months. In a multicenter study published in 2016 [18], CS demonstrated superiority versus celecoxib at reducing cartilage volume loss in knee osteoarthritis as measured by MRI. These results are in accordance with two previously published meta-analyses. Lee et al. [51] demonstrated that CS had a small but significant protective effect on minimum joint space narrowing after two years (SMD [standard mean difference] 0.261, 95% CI 0.131-0.392, P < 0.001). Hochberg [52] also demonstrated a small but significant effect of CS on the reduction in the rate of decline in minimum joint space width of 0.13 mm [95% confidence interval (CI) 0.06, 0.19] (P = 0.0002), which corresponded to a small effect size of 0.23 (95% CI 0.11, 0.35) (P = 0.0001). However, these conclusions were based on only three RCTs. Despite this, the results from these meta-analyses show that CS may delay the progression of OA in the knee after daily usage for two years.

CS origin and purity

CS is usually derived from bovine, porcine, chicken, and fish cartilage by extraction and purification processes. Natural-occurring CS has a molecular weight (MW) of 50-100 kDa; however, CS extraction reduces the MW to about 10-40 kDa [53]. Purification is important to minimize contaminants, such as other glycosaminoglycans, proteins, organic molecules, viruses, prions, and solvents [54].

Different sources of CS are postulated to have different compositions and potencies [55]. A 2013 study demonstrated that bovine-derived CS was the most effective in suppressing osteoclast activity, while fish and porcine CS were less consistent in their effects [56]. Bovine CS was also found to suppress IL-6 and PGE2 production, and effects were greater in the higher purity samples (99,9%), while other sources were less effective [57]. In a pharmacoproteomic study [58], lower-purity samples of porcine origin were shown to be pro-inflammatory, while CS of bovine origin with high purity was found to be anti-inflammatory and induced anabolic responses. This suggests that differences in the structure and composition of CS compounds can influence their pharmacological activity.

These results demonstrate the importance of choosing high-quality CS products for the treatment of osteoarthritis, as the source material and purification procedures might influence their therapeutic effects.

Chondroitin sulfate safety

Chondroitin sulfate is extracted from animal sources and submitted to purification processes for commercial use [53]. Due to different purification processes, the presence of bacteria, viruses, or prions cannot be excluded [59]. Additionally, various other natural contaminants may be present in CS products [12,53].

Due to extraction methods, CS products may also be contaminated with other polysaccharides that are simultaneously extracted with CS, such as hyaluronic acid, dermatan sulfate, and keratan sulfate [60]. The latter has been found to have the capacity to develop immunologic reactions [61].

Studies have also found that variable amounts of protein and nucleic acids may also be co-purified with CS. These proteins may also be a source of allergic and immunologic reactions [12].

Contamination with infective agents is also a possible source of severe adverse events, such as the transmission of prions and the possible development of Creutzfeldt-Jakob disease. As such, destruction of these infective agents by specific chemical steps is necessary to ensure safety [62].

Despite this, a recent meta-analysis provided strong evidence of the safety of CS supplements [63]. Another review that analyzed the safety of CS has found that the incidence of adverse events is low and similar to placebo, with gastrointestinal symptoms being the most common [64].

Chondroitin sulfate: new trials and future direction

More recently, investigations have focused on the anti-inflammatory properties of CS. In one recent study [65], CS induced the epithelial-mesenchymal transition, increased the expression of type II collagen and tissue inhibitors of metalloproteinases -1 and -2, and inhibited the expression and activity of metalloproteinase-9 and metalloproteinase-2. The phosphorylation of Akt, IκB kinase (IKK), IκB, and p65 was decreased by CS. CS treatment also resulted in β-catenin production, an important protein for maintaining cartilage homeostasis.

In another study [66], a semi-synthetic CS (CS-semi5) was synthesized and was shown to have positive effects on synovial inflammation, cartilage erosion, and bone loss in rheumatoid arthritis animal models. Another recent study [67] has also shown that CS E disaccharide has high anti-complement activity and anti-inflammatory properties for the treatment of OA through regulation of the complement system.

Other studies have focused on the chondroprotective effect of CS from different sources. In one particular study [68], microbial CS was produced from bacterial sources using biotechnological methods and showed promise as an alternative agent in the treatment of osteoarthritis.

At last, CS has been used as a delivery vehicle for other substances into the cartilage [69,70], which can signify a potential advance for the treatment of OA. In one study [69], chondroitin sulfate microspheres anchored with drug-loaded liposomes were developed to delay the progression of osteoarthritis through dual antioxidation. In another study [70], cassic acid-integrated nanoreservoirs (CA-NRs) were fabricated based on ionic conjugation between CA and the cationic hydrophobic surface modifier octadecylamine and further functionalized with CS to develop CS-CA-NRs. These CS-CA nanoreservoirs showed superior antiosteoarthritic activity in osteoarthritic mice, exhibiting higher cartilage repair compared to CA-NRs alone. Additionally, they also significantly inhibited OA inflammatory cytokines, degradation enzymes, and oxidative stress and improved cartilage matrix biosynthesis.

Discussion

This review of the literature on CS supplements for the treatment of OA revealed a complex picture regarding their therapeutic effects. As described previously, many biological effects have been demonstrated on articular tissues, including the stimulation of the synthesis of proteoglycans, collagen type 2, and hyaluronic acid and the inhibition of IL-1β and PGE2 [31-33]. Chondroitin sulfate also inhibits p38 MAPK, JNK, and Erk1/2, reduces metalloprotease activity, and limits NF-κB translocation [30]. By stimulating the synthesis of important components of healthy cartilage and limiting the activity of inflammatory mediators and signaling, chondroitin sulfate may have a role in protecting healthy cartilage from degeneration. However, the anti-inflammatory properties of CS are still under investigation, although the results from newer studies on this matter have been promising [64-68].

Excessive angiogenesis can promote inflammation and cartilage damage in the joints. Inhibiting inflammation and angiogenesis may provide effective therapeutics for the treatment of OA by improving symptoms and retarding joint damage. Studies have demonstrated that CS may have antiangiogenic properties, possibly by reversing the inhibitory effect of IL-1β on anti-angiogenic factors [42]. This anti-angiogenic effect may be important for the prevention of joint damage; however, more studies are necessary to verify this effect. CS has also shown antioxidant properties by reducing reactive oxygen species; this may reduce oxidative stress-induced damage to joints, which may contribute to osteoarthritis [41].

CS has also been shown to act as a SYSADOA by reducing symptoms of pain and improving function, and possibly as a DMOAD by limiting cartilage volume loss and joint space narrowing [11-13]. These effects suggest that CS products may be important to enhance the patient's quality of life by alleviating the severe symptoms of the disease. However, the optimal duration of treatment and CS dosage are still uncertain, although a meta-analysis concluded that larger dosages of 1200 mg/d appear to have more benefit than lower dosages [48]. In regard to stopping OA progression, evidence has been conflicting [18,49,50,52], and there is still a need for further investigation to establish CS efficacy as a DMOAD.

For further research on CS therapeutic efficacy, the choice of the CS product is essential, as different sources and purification methods can impact the pharmacological activity of CS. As has been shown in this review, the purity of CS products, even among pharmacological-grade products, varies considerably from the percentages described on the label [45]. The choice of high-grade pharmacological CS products is necessary when studying their effects on OA since these products have shown more consistent therapeutic effects compared to CS food supplements [24]. Many of these food supplements do not meet label specifications and may contain low-quality ingredients. There are also concerns about the contamination of CS supplements with by-products of purification methods and possible contamination with infective agents, which could have serious consequences for the patients [59]. We conclude that only high-grade pharmacological CS supplements should be used for clinical use and clinical trials.

More recently, CS has been trialed as a delivery mechanism for other substances into the cartilage. This type of combination therapy may be important in delaying the progression of OA. In some animal studies, promising results have been achieved in this regard [69,70].

## Conclusions

In conclusion, the investigation of CS use for the treatment of OA is still ongoing. This review offers insight into many of its therapeutic effects and current trials on the topic; however, study methodology flaws and lesser-quality products may have limited reaching definitive conclusions on this matter in the past. The concurrent use of glucosamine sulfate and chondroitin sulfate in many of these trials may have also limited the differentiation of the isolated benefit of CS. In the future, further investigation should strive to use CS supplements of pharmacologic grade that have been properly subjected to rigorous purification methods and quality control. Study protocols should also be properly standardized, with well-designed randomized controlled trials and a longer-term follow-up that is needed to derive benefit from this slow-acting drug. Newer studies should also strive to use CS supplements as monotherapy, avoiding combinations with other SYSADOAs such as glucosamine.

In brief, this review emphasizes the importance of only using high-quality CS products when conducting clinical trials and for clinical use to guarantee the optimal management of osteoarthritis.
